# Premature mortality attributable to smoking among Tunisian men in 2009

**DOI:** 10.18332/tid/112666

**Published:** 2019-11-04

**Authors:** Olfa Saidi, Said Hajjem, Nada Zoghlami, Hajer Aounallah-Skhiri, Nadia Ben Mansour, Mohamed Hsairi, Habiba Ben Romdhane, Julia A. Critchley, Dhafer Mallouche, Martin O’Flaherty, Radhouane Fakhfakh

**Affiliations:** 1 National Institute of Public Health, Ministry of Health, Tunis, Tunisia; 2 Research Laboratory of Epidemiology and Prevention of Cardiovascular Diseases, Faculty of Medicine, University Tunis El Manar, Tunis, Tunisia; 3 National Health Institute, Tunis, Tunisia; 4 Faculty of Medicine of Tunis, University of Tunis El-Manar, Tunis, Tunisia; 5 The SURVEN (Nutrition Surveillance and Epidemiology in Tunisia) Research Laboratory, National Institute of Nutrition and Food Technology, Tunis, Tunisia; 6 Salah Azaiez Institute, Tunis, Tunisia; 7 Division of Population Health Sciences and Education, St George’s, University of London, London, United Kingdom; 8 National Institute of Statistics and Data Analysis, Tunis, Tunisia; 9 Department of Public Health and Policy, University of Liverpool, Liverpool, United Kingdom; 10 Unit of Research in Tobacco Epidemiology and Control, Tunis, Tunisia

**Keywords:** premature mortality, tobacco use, Tunisia

## Abstract

**INTRODUCTION:**

Tobacco smoking is a significant public health threat in the world, a risk factor for many diseases, and has been increasing in prevalence in many developing countries. In this study, we aimed to estimate the burden of premature deaths attributable to smoking among Tunisian men aged 35–69 years in 2009.

**METHODS:**

The number of deaths attributable to smoking was estimated using the population attributable risk fraction method. Smoking prevalence was obtained from a nationally representative survey. Causes of death were obtained from the registry of the National Public Health Institute. Relative risks were taken from the American Cancer Society Prevention Study (CPS-II).

**RESULTS:**

Total estimated premature deaths attributable to smoking among men in Tunisia were 2601 (95% CI: 2268–2877), accounting for 25% (95% CI: 23.3–26.6) of total male adult mortality. Cancer, cardiovascular and respiratory diseases were the major causes of premature deaths attributable to smoking with 1272 (95% CI: 1188–1329), 966 (95% CI: 779–1133) and 364 (300–415) deaths, respectively.

**CONCLUSIONS:**

Tobacco smoking is highly relevant and is related to substantial premature mortality in Tunisia, around double that estimated for the region as a whole. This also has not decreased over the past 20 years. Urgent actions are needed to reduce this pandemic.

## INTRODUCTION

Tobacco smoking has been estimated to be the second leading risk factor for death from all causes^1-3^, and it is the leading cause of preventable death (death at ages <70 years) in the world. According to the World Health Organization (WHO), tobacco use results in about 6 million deaths per year and this number will increase to more than 8 million per yearleading risk factor for death from all causes^1-3^, and results in about 6 million deaths per year and this it is the leading cause of preventable death (death number will increase to more than 8 million per year by 2030. More than 80% of those deaths will occur in low- and middle-income countries^4^, and many at early ages. The number of premature deaths (death at ages 30–69 years) was estimated at 4·83 million in 2000. Ezzati et al.^3^ estimated that only half of these deaths were registered in developing countries. The number of smoking-attributable deaths will increase considerably in these countries during the next few decades^3^. In 2010, 8.4% of the worldwide burden of diseases in men were attributed to tobacco smoking (including secondhand smoke), which made smoking the leading risk factor for death in men, while this proportion was 3.7% in women (fourth highest risk factor)^5^. According to the Global Burden of Disease group, smoking was the fourth most crucial risk factor in terms of Disability-Adjusted Life Years (DALYs) after dietary risks, high blood pressure and high body mass index^6^, in Tunisia.

In many developed countries, the proportion of deaths attributed to smoking increased from 10.3% to 22.3% in men, and from 0.7% to 8.1% in women between 1955 and 2000^7^. In Tunisia, smoking prevalence in men has been very high for some time. In 1997, 30.5% of adults were current smokers (55.8% in men and 5.3% in women), while more recent studies have confirmed that these smoking rates remain broadly constant in men (55.2%)^8,9^. The burden of deaths attributable to smoking in men in Tunisia is hence likely to be high, but no estimate of this has been made since 1997^10,11^. The purpose of our study was to estimate the burden of premature deaths attributable to smoking among Tunisian men aged 35–69 years in 2009.

## METHODS

The number of deaths was estimated using the WHO methodology, which requires data on smoking prevalence, causes of deaths, population size and relative risks^12^ (Table 1).

**Table 1:** Population sizes, number of deaths and smoking prevalence in men aged 35–69 years in Tunisia, 2009

*Age group (years)*	Non-smokers* %	Former smokers* %	Current smokers* %	Population** ( 1000 )	Deaths** n
35–39	26.2	14.7	59.1	347	587
40–44	29.8	17.8	52.5	340	809
45–49	29.0	18.4	52.6	320	980
50–54	30.0	26.5	43.4	280	1546
55–59	21.6	31.3	47.1	208	1937
60–64	34.9	25.7	39.4	138	1977
65–69	35.5	37.3	27.3	122	2613
**Total**	**29.0**	**22.3**	**48.7**	**1756**	**10449**

Sources: * TAHINA Survey, **National Institute of Statistics.

### Smoking prevalence

Smoking prevalence was obtained from the Epidemiological Transition and Health Impact in North Africa (TAHINA) survey. This was conducted in Tunisia in 2005 and included a nationally representative sample of 8007 individuals, aged 35–70 years^13^. ‘Smoker’ was defined as someone who reported smoking cigarettes daily for at least the month previous to the survey. ‘Former smokers’ were defined as adults who have smoked in their lifetime but report that they do not currently smoke. We assumed that smoking prevalence in 2009 would have been approximately the same as in 2005. This assumption seems reasonable since smoking prevalence in adult men has remained mostly constant for the past ten years^9^.

### Causes of death

Tunisian population estimates and number of deaths (distributed by age and sex) were obtained from the National Institute of Statistics^14^; while causes of death in 2009 were obtained from the Tunisian causes of death surveillance system (National Institute of Public Health, Ministry of Public Health), which uses the international death certificate model . Causes of death were coded according to the 10th revision of International Classification of Diseases (ICD10), using the STYX Software^15^. All deaths are recorded in Tunisia, and cause of death confirmed by a clinician is available for about 50% of the total. Based on the ANACONDA tool applied to Tunisian data in 2013, the weighted scores of the quality of cause of death reporting, quality of age and sex reporting, the level of cause-specific detail available, are 72.2, 91.3 and 90.3, respectively^16^.

We assumed that the causes of death by age were the same amongst those deaths with and without a classified cause. We applied the per cent of causes of deaths by gender and age groups to the total deaths registered in Tunisia. Premature mortality was defined as deaths occurring in the age group 35–69 years.

### Population data

The Tunisian male population aged ≥35 years was obtained from Tunisian National Institute of Statistics for 2009. In 2009, the Tunisian population was approximately 10 million, and there were 1.756 million men aged 35–69 years (33.7% of the total population of men). In this same year, there were 10449 deaths among men aged 35–69 years^14^.

### Relative risks and mortality attributable to smoking

Relative risks for specified causes of death among both current and former smokers of cigarettes, for men aged ≥35 years, were taken from the American Cancer Society Prevention Study (CPS-II)^17^ (Table 2).

**Table 2:** Mortality attributable to smoking in men in Tunisia, 2009

*Diseases*	*%*	*Deaths*	*Current Smoker*	*Former smoker*	*Smoking attributable mortality*	*Proportion attributable*
		*n*	*RR*	*RR*	*n (95% CI)*	*n (95% CI)*
**Cardiovascular**						
Chronic heart	10.1	1057	1.94	1.41	375 (327–418)	35.4 (30.9–39.5)
Cerebrovascular	7.4	777	2.24	1.29	311 (236–378)	40.1 (30.3–48.6)
Other circulatory	0.5	53	4.06	2.33	34 (29–39)	64.1 (53.9–72.2)
Other heart	7.2	752	1.85	1.32	246 (188–299)	32.7 (24.9–39.7)
All	25.3	2639	–		966 (779–1133)	-
**Respiratory**						
Chronic obstructive	2.7	281	9.65	8.75	241 (226–252)	85.6 (80.3–89.5)
pulmonary						
Other	3.1	325	1.99	1.56	123 (74–164)	37.8 (22.7–50.4)
All	5.8	606	–		364 (300–415)	-
**Cancers**						
Lip, oral cavity, pharynx	1.0	105	27.48	8.80	98 (87–102)	93.6 (82.7–97.6)
Esophagus	0.3	36	7.60	5.83	29 (23–32)	81.1 (64.1–90.2)
Pancreas	1.1	120	2.14	1.12	44 (25–60)	36.8 (20.5–50.16)
Larynx	0.3	27	10.48	5.24	23 (16–26)	84.8 (58.8–94.63)
Lung	10.7	1118	22.36	9.36	1034 (1012–1052))	92.5 (90.5–94.0)
Kidney	0.0	0	2.95	1.95	0	–
Bladder, other urinal	0.8	83	2.86	1.90	44 (26–57)	52.5 (31.5–67.8)
All	14.3	1490			1272 (1188–1329)	-
**All causes**		**4735**			**2601 (2268–2877)**	

RR: Risk Ratio.

The number of deaths was estimated using population attributable risk fraction, by specific causes and age groups, based on Levin’s formula^18^. Calculations were performed in MS Excel (Table 2). For sensitivity analyses, we used the upper and lower bounds of the 95% confidence intervals (CIs) for the prevalence of current and former smoking (from our own TAHINA surveys) and upper and lower values for relative risk (obtained from the US CPS-II dataset).

## RESULTS

**Figure 1:**
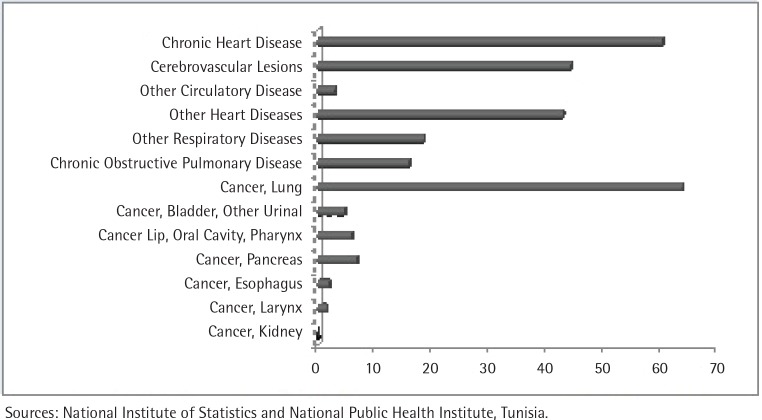
Mortality rates per 100000 by causes among men aged 35-69 years in 2009

**Figure 2:**
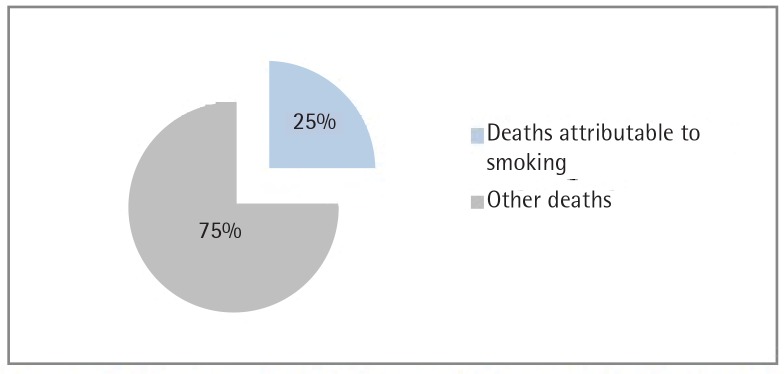
Deaths attributable to smoking among men aged 35-69 years in Tunisia in 2009

In 2009, the crude mortality rate was 595 per 100000 among men aged 35–69 years. Figure 1 summarizes the leading causes of premature mortality among men in Tunisia, which were lung cancer (64 per 100000) followed by chronic heart diseases (60 per 100000) and cerebrovascular disorders (44 per 100000). The estimated number of premature deaths attributable to smoking among men was 2601 (95% CI: 2268–2877), corresponding to 25% (95% CI: 23.3–26.6) of the total number of premature deaths for men (Figure 2). Cancer, cardiovascular disease and respiratory diseases were the major causes of premature death attributable to smoking accounting for 1272 (95% CI: 1188–1329), 966 (95% CI: 779–1133) and 364 (95% CI: 300–415) deaths, respectively [48.9% (95% CI: 46.2–51.7), 37.1% (95% CI: 34.4–40.4) and 14.0% (11.8–16.2)], as outlined in Table 2.

### Deaths attributable to smoking applying a sensitivity analysis (minimum-maximum)

In 2009, 93.6% (95% CI: 82.7–97.6) of deaths caused by lip, oral cavity and pharynx cancers among men aged 35–64 years were attributable to smoking. Lung cancer had a high attributable risk associated with smoking among men [92.5% (95% CI: 90.5–94.0) of lung cancer deaths], the chronic heart disease [35.4% (95% CI: 30.9–39.5) of chronic heart disease deaths], cerebrovascular disorders [40.1% (95% CI: 30.3–48.6) of cerebrovascular disorders deaths] and chronic obstructive pulmonary disease [85.6% (95% CI: 80.3–89.5)]. These diseases accounted for approximately 76.0% of total premature deaths attributable to smoking among men (Table 2).

## DISCUSSION

This study showed that tobacco smoking in 2009 was responsible for about a quarter (2601 deaths) of premature deaths among Tunisian men. This could well confirm the propagation of the tobacco epidemic for men in Tunisia, referring to the Lopez et al.^19^ propagation model of tobacco use epidemic. A recent study advocates considering the tobacco epidemic separately for men and women, particularly in developing countries^20^, since the factors that lead to the start and continuation of cigarette smoking and tobacco use may be different for women than for men, and may also differ between developed and developing countries. However, in fact, the smoking status of women in Tunisia seems to be almost similar to that in Western countries during the middle of 20th century with a substantial decrease in societal and cultural prohibition of cigarette smoking among women.

These estimates for 2009 were slightly lower than the previous ones of 1997 in Tunisia reported by Fakhfakh et al.^10^ who estimated that 6435 deaths occurred because of tobacco smoking in adults, amounting to 22% of total male deaths. However, the comparison of the two studies must be interpreted with caution due to methodological differences. The earlier study used the distribution of deaths by causes published by WHO in 1998 since the Tunisian information system on causes of death was not available at that time. Moreover, the earlier study included younger men aged >25 years, and all tobacco types (the use of other types of tobacco in Tunisia is about 1.5%)^10^. Nevertheless, our calculated proportion of smoking-attributable mortality was over twice that estimated by WHO for the Eastern Mediterranean region (12% for adult men), and it also exceeds that of China (12.9% among men)^21^, but not of western countries such France (33%). Our estimate is close to some low- and middle-income countries such as India (20% for men aged 30–69 years in 2010)^12,22,23^.

Our results established that the leading causes of premature deaths from smoking were cancers, cardiovascular diseases and respiratory diseases. This is in line with findings from other studies, e.g. US in 2004^24^. Ezzati and Lopez^3^ also estimated that lung cancer was the disease with the highest fraction attributable to smoking worldwide in 2000. Almost three quarters (71.0%) of all lung cancers or 0·85 million deaths (79.0% or 0·69 million deaths among men and 48% or 0·16 million deaths among women) were attributable to smoking^7^. Thus, lung cancer mortality is the most specific indicator of tobacco smoking effects on health at the population level^25^. Cancer is the second leading cause of death globally, with about 70% occurring in low- and middle-income countries^26^. The most common is lung cancer^27^. Due to reduced survival, the main burden of lung cancer is due to premature mortality rather than long-term illness. Lung cancer was the leading cause of cancer-related premature mortality among males (20%)^28^.

In comparison, in Eastern Morocco, lung cancer ranked the fourth most common cancer, accounting for 7.5% of total cancers and the most frequent malignancy with 19% of male cancers^29^. In Algeria, lung cancer is the sixth most common type of cancer (2.8%), and it is the most prevalent cancer in males representing 19.9% of cases^30^.

About one-third of deaths from cancer are due to the 5 leading behavioral and dietary risks: tobacco use is the most critical risk factor for cancer and is responsible for approximately 22% of cancer deaths^31^. The mortality rate of deaths from lung cancer almost doubled from 34.8 per 100000 to 58.8 per 100000 among Tunisian men between 1997 and 2009 (reflecting the alarming situation of mortality and morbidity related to tobacco use in Tunisia)^9^. Unless steps are taken to reduce cigarette smoking, the coming decades will experience a significant increase in smoking related mortality and morbidity. Consequently, the economic burden will be high both for the community and also for many of the poorer households^32^. Since tobacco-induced diseases such as cancers and cardiovascular diseases are costly, out-of-pocket expenditure on health care is high in Tunisia.

In 2010, Tunisia adopted WHO Framework Convention on Tobacco Control, and a strategy was implemented in 2009, with clear goals, but still modest results, due to difficulties with implementation^33^. Since around 1992, several anti-smoking measures commenced in Tunisia including information campaigns and passing of an anti-smoking law to restrict smoking in public places; a decrease in tobacco sales has been observed but no decrease in smoking prevalence. It is thought that more smokers are purchasing cigarettes illegally, with substantial increases in tobacco smuggling due to political and security problems in Tunisia over the past three years. Moreover, smoking cessation interventions should be more targeted to high-risk groups to be more effective, such as young smokers and females, where smoking prevalence trends are highly worrisome^34,35^.

In this study, reliable data were used. Mortality data were obtained from registry of deaths collected by the other estimates, e.g. those produced by WHO EMRO rely on estimated mortality obtained from other countries with similar standards of living and development in the Region. This paper is the first to use Tunisian data from the national registry that records the death certificates. A recent assessment by WHO estimated that this mortality database was 87% accurate^36^. Demographic information was provided from the census data while the relative risks were obtained from CPS-II, an extensive prospective cohort study, used in the majority of studies on smoking-attributable mortality carried out in both developed and developing countries^37^. CPS-II relative risks were calculated in a smoking population consuming on average 20 cigarettes per day^7^, which is similar to the Tunisian context (the average daily consumption per men was 18±9 cigarettes in 1997^8^ and 20±12 in 2005^13^). Applying CPS-II relative risks is considered appropriate in the absence of extensive prospective studies from Tunisia or the Middle East and North Africa region (MENA region).

### Strengths and limitations

The key strengths of this analysis include the nationally representative data collected through TAHINA. Conversely, this study had some limitations. The first is related to the fact that in this article, smoking prevalence excluded secondhand smoke and other types of tobacco use, especially waterpipe and chewing tobacco. These types of tobacco use are relatively common in Tunisia^38^, but their health impacts and relative risks poorly studied and documented^39^. This exclusion suggests that the estimate of tobacco attributable mortality may be even higher in Tunisia. Most studies of mortality attributable to tobacco have only included cigarette smoking and ignored other types of tobacco use, except in the Indian Subcontinent where smokeless tobacco is particularly common^40^. Another limitation of this study is that we excluded liver and stomach cancers in our estimation. Latency may be a limitation, as smoking in men in Tunisia has been constant for some time and all causes of death that might possibly be attributed to smoking were not included.

Another limitation is that we focused on only mortality attributable to smoking in men, excluding women, even though female smoking is thought to be increasing. Unfortunately, there are no data considered sufficiently reliable to estimate the prevalence of smoking in Tunisian women. The current national estimate of 4% is likely to be a substantial underestimate^6^. This is because female smoking is still culturally prohibited^13^. For example, the association between lung cancer in women and tobacco smoking differs between countries. The highest estimates come from North American studies, with relative risk near 20, though the lowest come from Asian studies^24^.

## CONCLUSIONS

Cigarette consumption in Tunisian men was responsible for 2601 deaths in 2009, one-quarter of premature deaths. These estimates of premature mortality have significant public health implications. The tobacco epidemic in Tunisia is firmly established and results in an unavoidable burden of morbidity and mortality in Tunisia. In regard to this alarming situation, effective interventions to reverse this trend were already taken a few years ago; Tunisian decision-makers defined a strategy in 2009 to reduce smoking prevalence, tobacco control actions involving legislation, education at worksites and schools, and the implementation of outpatient smoking cessation programs in health centers, but this has not been enforced. Now, more focus must be given to stop the flow of new smokers, from population groups such as youth and females.

## CONFLICTS OF INTEREST

The authors have completed and submitted the ICMJE Form for Disclosure of Potential Conflicts of Interest and none was reported.
